# The Perspectives Associated With the Computer-Based Diagnostic Method of Depressive Disorder

**DOI:** 10.3389/fpsyt.2018.00687

**Published:** 2018-12-19

**Authors:** Elena Bartkiene, Vesta Steibliene, Virginija Adomaitiene, Vita Lele, Darius Cernauskas, Daiva Zadeike, Dovile Klupsaite, Grazina Juodeikiene

**Affiliations:** ^1^Department of Food Safety and Quality, Lithuanian University of Health Sciences, Kaunas, Lithuania; ^2^Psychiatric Clinic, Lithuanian University of Health Sciences, Kaunas, Lithuania; ^3^Department of Food Science and Technology, Kaunas University of Technology, Kaunas, Lithuania; ^4^Food Institute, Kaunas University of Technology, Kaunas, Lithuania

**Keywords:** depressive disorder, food, food taste, emotions, FaceReader, computer based diagnostic

## Abstract

Depressive disorder (DD) shortens a healthy and productive human life, has significant public health costs is and associated with high suicide rates. In depression sadness and emotional misery manifest in facial expressions, as psychomotor slowing, lack of energy, high tension, and attenuated sensory perception. Loss of appetite, changes to the taste of food, and the loss of pleasure in eating are important criteria in the diagnosis of DD. We hypothesized that a patient's facial expressions and emotional responses to different tastes can be used as the diagnostic moderators for the development of a new contactless, computer-based method for diagnosis of DD. The confirmation of this hypothesis can shed a new perspective on early contactless, computer-based psychiatric diagnostic strategies and early identification of DD symptoms, as DD is an important issue in public mental health. The benefits of this method are evidence from several perspectives (I) patients can use a self-rating instrument to assess DD symptoms; this may act as an incentive to seek professional help; (II) family and community can use an instrument for early recognition of DD symptoms and suicidal tendencies, making it possible to encourage the individual to seek professional health care; (III) general practitioners have a reliable instrument for preliminary diagnosis of DD in primary care, thus saving the time and resources; (IV) public health benefits include early diagnosis and treatment of DD and better outcomes, reductions in disability-adjusted life years and the global burden of the disease. It is nevertheless important to recognize the limitations and risks of contactless diagnosis of DD. As it is a self-assessment method it is not possible to rule out false positives and false negatives. However, this method might be used for early diagnosis of DD symptoms. Also, it should be mentioned that further evaluation and an experts opinion about this method is needed. The clinical diagnosis of DD should continue to be made by healthcare professionals. Finally, this method may perspectively predict DD at an early stage and may ensure a higher quality of the patients' primary care in the public health system.

Depressive disorder (DD) is a common, often progressive and recurrent disorder. If left untreated or treated inadequately it may become chronic and treatment-resistant, with a significant negative impact on everyday functioning. It shortens a healthy and productive human life, has significant public health costs and is associated with high suicide rates. According to the World Health According (WHO), DD affects more than 300 million people worldwide and close to 800,000 people die by suicide every year; the WHO estimates that by 2020 the prevalence of DD will reach 5.7% and it will have become the second most common cause of disability-adjusted life years worldwide (1). DD develops gradually and in many cases the sufferer does not recognize the initial symptoms and seeks help only when the disorder has become severe. Although effective treatments for DD are available, globally fewer than half of those affected (in many countries, fewer than 10%) receive such treatment (2). If DD remains untreated for a long period this has a negative impact on treatment effectiveness, the duration of the disorder, outcomes and survival of somatic comorbidities (3–5). DD is associated with an imbalance in neurotransmitters, pituitary-hypothalamic-adrenal axis dysfunction, and changes in levels of neurotrophic factors that are considered direct precursors of neurodegenerative disorders and dementia (6–9). In primary care DD often remains undiagnosed and untreated. Due to the stigma associated with the disease patients are often reluctant to seek psychiatric help and long waiting times and gaps in the healthcare system can also reduce access to treatment. Moreover, patients often do not report depressive symptoms to their family doctor; due to lack of time family doctors do not use screening instruments and often fail to recognize symptoms of DD or offer only inadequate treatment (10). This increases suicide rates dramatically (11).

Better understanding of the pathophysiology of DD has led to evaluation of different genetic, neuroimaging, and biochemical biomarkers and instruments for objective diagnosis of DD in clinical settings (12). Given the need for a reliable, brief, and easy-to-administer screening instrument to help diagnose DD, the development of a contactless, computer-based method of early diagnosis should be a priority (12).

There is a growing body of evidence on the influence of nutrition on mental health, particularly affective symptoms (13). It has been established that diet and individual food products influence the occurrence, onset, severity, and duration of DD (14). Newly developed effective public health nutritional strategies could be used to prevent clinical depression (15). Depression manifests as sadness, and emotional misery that is visible in facial expressions, as psychomotor slowing, low energy, high tension, and attenuated sensory perception. Loss of appetite, loss of sense of taste, and pleasure in eating are important DD diagnostic criteria, although atypical depression may be characterized by a strong preference for energy-dense foods, fatty foods, and sweet foods. Uncontrolled eating, satiation and altered food preferences as well as appetite may indicate negative mood (16). The mentioned changes could be due to the influence of carbohydrates and proteins on the tryptophan ratio in the brain, as well as to the effects of such changes on mood and arousal given that there could be individual differences in susceptibility to nutritional effects on mood, emotion and aspects of brain function (17).

It is known that most depressed patients show impaired production of emotional facial expressions, particularly positive expressions (18). It has been reported that affective facial reactions to the food tastes are important factors in food intake and even predict body mass index (19). Facial expressions and emotional response to difference tastes can be measured using *FaceReader 4* software (Noldus Information Technology, The Netherlands). The data obtained from the facial expression of emotions to the different food tastes have been shown to correlate with data of conventional sensory analysis (20). Our previous research showed that the evaluations of facial expressions obtained using FaceReader 4 software were strongly correlated with self-reported hedonic liking, indicating that computer-based technology can be a sufficiently accurate tool for the food tastes induced emotions evaluation (21).

On this basis we hypothesized that seven categories of emotional response (neutral; angry; disgusted; happy; sad; scared; surprised) to different tastes (neutral; acid; sweet; salt; bitter) would be strongly correlated with clinical symptoms of DD as evaluated using clinical diagnostic instruments and depression severity rating scales (Montgomery and Asberg Depression Rating Scale MADRS; (22)]. We also hypothesized that a patient's facial expression of emotions to different tastes of food can be used as a diagnostic moderator for the development of a new contactless, computer-based diagnostic method and support the creation of algorithm for DD diagnosis (Figure 1).

**Figure 1:**
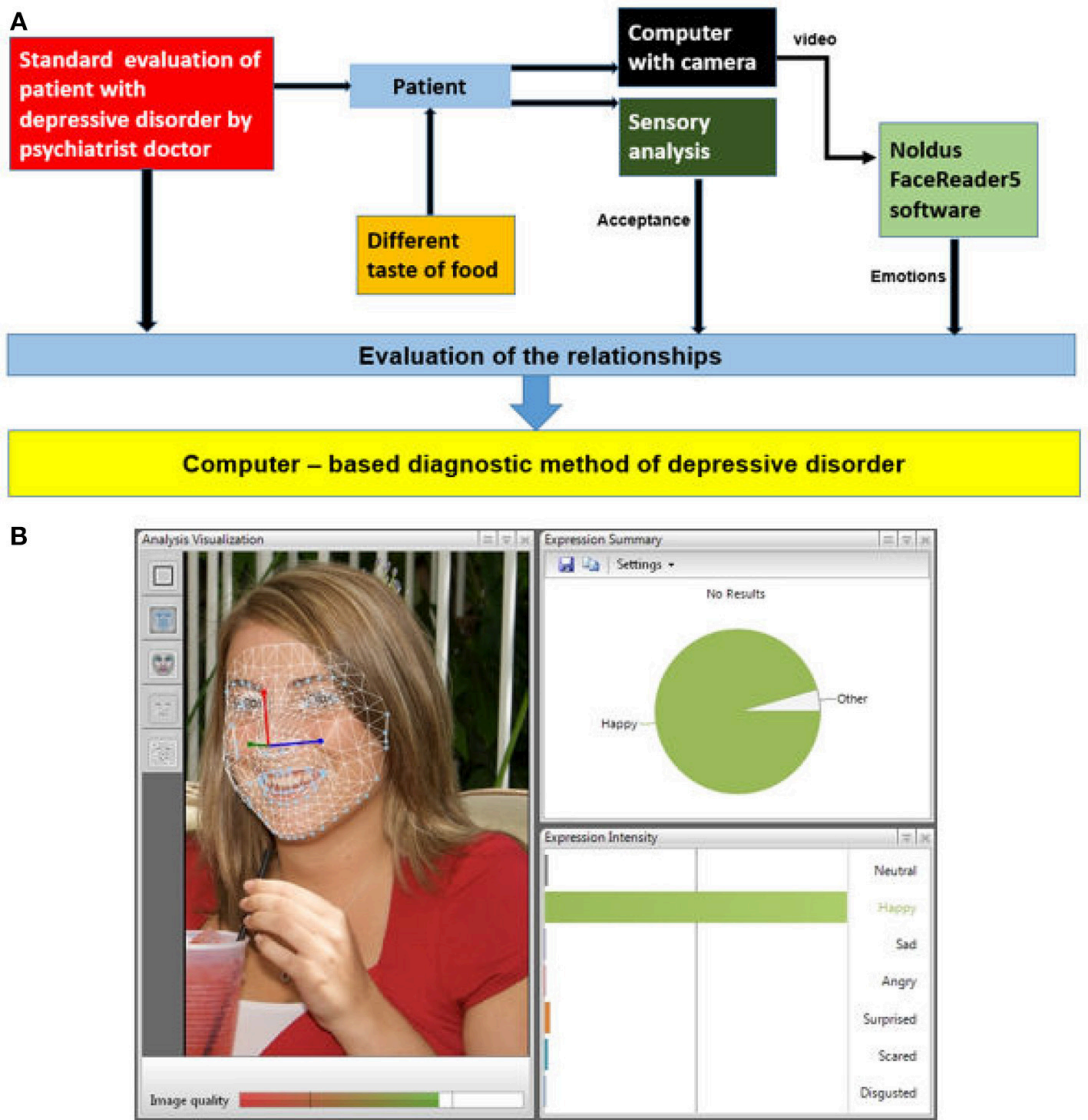
The main steps in the development of a computer-based method of diagnosing depressive disorder: **(A)** the main screen of *FaceReader*: bar graphs, line graphs, and pie chart; **(B)** original image.

The benefits of this method are evidence from several perspectives (I) patients can use a self-rating instrument to assess DD symptoms; this may act as an incentive to seek professional help; (II) family and community can use an instrument for early recognition of DD symptoms and suicidal tendencies, making it possible to encourage the individual to seek professional health care; (III) general practitioners have a reliable instrument for preliminary diagnosis of DD in primary care, thus saving the time and resources; (IV) public health benefits include early diagnosis and treatment of DD and better outcomes, reductions in disability-adjusted life years and the global burden of the disease.

It is nevertheless important to discuss the limitations and the risks of contactless diagnosis of DD. Since this is a self-assessment method it is not possible to rule out false positives and false negatives.

However, this method might be used for early diagnosis of DD symptoms. Also, it should be mentioned that further evaluation and experts opinion regarding relevance and accuracy of this method is needed. The clinical validation of DD diagnosis should be performed by healthcare professionals. Finally, this method may perspectively predict DD at an early stage and may ensure a higher quality of the patients' primary care in the public health system.

## Author Contributions

EB and VS designed the study and wrote up the initial draft. VA and GJ are principle supervisors overseeing the study. VL, DZ, DK, and DC were involved with the design of the study and the drafting of the paper.

### Conflict of Interest Statement

The authors declare that the research was conducted in the absence of any commercial or financial relationships that could be construed as a potential conflict of interest.
