# Corrigendum: The Perspectives Associated With the Computer-Based Diagnostic Method of Depressive Disorder

**DOI:** 10.3389/fpsyt.2019.00012

**Published:** 2019-01-29

**Authors:** Elena Bartkiene, Vesta Steibliene, Virginija Adomaitiene, Vita Lele, Darius Cernauskas, Daiva Zadeike, Dovile Klupsaite, Grazina Juodeikiene

**Affiliations:** ^1^Department of Food Safety and Quality, Lithuanian University of Health Sciences, Kaunas, Lithuania; ^2^Psychiatric Clinic, Lithuanian University of Health Sciences, Kaunas, Lithuania; ^3^Department of Food Science and Technology, Kaunas University of Technology, Kaunas, Lithuania; ^4^Food Institute, Kaunas University of Technology, Kaunas, Lithuania

**Keywords:** depressive disorder, food, food taste, emotions, FaceReader, computer based diagnostic

In the published article, there was an error regarding the affiliations for Darius Cernauskas. As well as having affiliation 3, they should also have “Food Institute, Kaunas University of Technology, Kaunas, Lithuania.”

The authors apologize for this error and state that this does not change the scientific conclusions of the article in any way. The original article has been updated.

